# Whole-miRNome sequencing: a panel for the targeted sequencing of all human miRNA genes

**DOI:** 10.1093/nar/gkaf812

**Published:** 2025-08-27

**Authors:** Paulina Galka-Marciniak, Martyna Olga Urbanek-Trzeciak, Daniel Kuznicki, Natalia Szostak, Adrian Tire, Paulina Maria Nawrocka-Muszynska, Katarzyna Chojnacka, Malwina Suszynska, Katarzyna Klonowska, Karol Czubak, Magdalena Machowska, Anna Philips, Konstantin Maksin, Laura Susok, Michael Sand, Janusz Rys, Jolanta Jura, Magdalena Ratajska, Hanna Dams-Kozlowska, Janusz Kowalewski, Marzena Anna Lewandowska, Piotr Kozlowski

**Affiliations:** Institute of Bioorganic Chemistry, Polish Academy of Sciences, 61-704 Poznan, Poland; Institute of Bioorganic Chemistry, Polish Academy of Sciences, 61-704 Poznan, Poland; Institute of Bioorganic Chemistry, Polish Academy of Sciences, 61-704 Poznan, Poland; Institute of Bioorganic Chemistry, Polish Academy of Sciences, 61-704 Poznan, Poland; Institute of Bioorganic Chemistry, Polish Academy of Sciences, 61-704 Poznan, Poland; Institute of Bioorganic Chemistry, Polish Academy of Sciences, 61-704 Poznan, Poland; Institute of Bioorganic Chemistry, Polish Academy of Sciences, 61-704 Poznan, Poland; Institute of Bioorganic Chemistry, Polish Academy of Sciences, 61-704 Poznan, Poland; Institute of Bioorganic Chemistry, Polish Academy of Sciences, 61-704 Poznan, Poland; Institute of Bioorganic Chemistry, Polish Academy of Sciences, 61-704 Poznan, Poland; Laboratory of Experimental Medicine, Medical University of Warsaw, 02-091 Warsaw, Poland; Institute of Bioorganic Chemistry, Polish Academy of Sciences, 61-704 Poznan, Poland; Laboratory of Nuclear Proteins, Faculty of Biotechnology, University of Wroclaw, 50-383 Wroclaw, Poland; Institute of Bioorganic Chemistry, Polish Academy of Sciences, 61-704 Poznan, Poland; Medical Department, University of Commerce and Services (WSHIU), 61-485 Poznan, Poland; Department of Dermatology, Venereology and Allergology, Ruhr University Bochum, 44801 Bochum, Germany; Department of Dermatology, Dortmund Hospital gGmbH and Faculty of Health, Witten/Herdecke University, 44137 Dortmund, Germany; Department of Dermatology, Venereology and Allergology, St. Josef Hospital, Ruhr University Bochum, 44791 Bochum, Germany; Department of Plastic, Reconstructive and Aesthetic Surgery, St. Josef Hospital, 45239 Essen, Germany; Maria Sklodowska-Curie National Research Institute of Oncology, 31-115 Krakow, Poland; Department of General Biochemistry, Faculty of Biochemistry, Biophysics and Biotechnology, Jagiellonian University, 31-007 Krakow, Poland; Division of Pathology and Neuropathology, Medical University of Gdansk, 80-210 Gdansk, Poland; Department of Medical Laboratory Science, University of Otago, 9016 Dunedin, New Zealand; Department of Cancer Immunology, Poznan University of Medical Sciences, 61-701 Poznan, Poland; Department of Diagnostics and Cancer Immunology, Greater Poland Cancer Centre, 61-866 Poznan, Poland; The Ludwik Rydygier Collegium Medicum, Department of Thoracic Surgery and Tumors, Nicolaus Copernicus University, 85-067 Bydgoszcz, Poland; The Ludwik Rydygier Collegium Medicum, Department of Thoracic Surgery and Tumors, Nicolaus Copernicus University, 85-067 Bydgoszcz, Poland; The Franciszek Lukaszczyk Oncology Center, Department of Molecular Oncology and Genetics, 85-796 Bydgoszcz, Poland; Institute of Bioorganic Chemistry, Polish Academy of Sciences, 61-704 Poznan, Poland

## Abstract

Interest in the genetic variation of noncoding genomic elements, including microRNAs (miRNAs), is growing, and several mutations in miRNA genes implicated in human diseases, including cancer, have already been detected. However, the lack of dedicated analytical tools severely hampers progress in this area. In this study, we developed the first whole-miRNome sequencing (WMS) platform, which enables the targeted sequencing of all human miRNA genes (*n* ∼2000) and 28 miRNA biogenesis genes. By sequencing various types of DNA samples, including ∼300 tumor/normal pairs, from lung, colorectal, ovarian, renal, and basal cell carcinomas, we identified ∼2000 mutations, including 879 in miRNA genes. These mutations were located in all parts of the genes, including seed or cleavage sites essential for the functioning of miRNA genes. The high reliability of the mutations was confirmed through various approaches, including different sequencing methods. The analysis identified several miRNA genes with functional enrichment of cancer mutations, including *MIR3928*, which was specifically mutated in basal cell carcinoma, suggesting its potential role in this cancer. WMS also allowed the identification of multiple copy number alterations, which often encompassed miRNA genes. WMS provides highly effective, low-cost sequencing of all miRNA genes in different types of samples, including highly degraded ones.

## Graphical Abstract

**Graphical Abstract:**
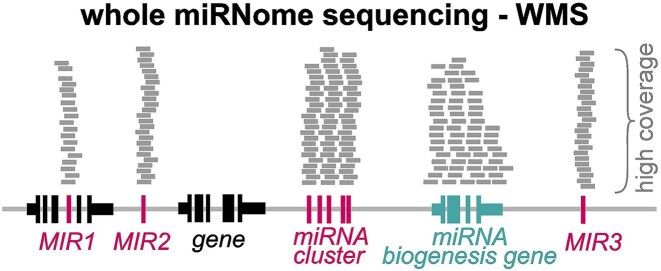

## Introduction

microRNAs (miRNAs) are short (19–23 nt), single-stranded, noncoding RNAs that post-transcriptionally downregulate the expression of the majority of human genes by translational repression and/or mRNA deadenylation and degradation. Over three decades of research have shown that specific miRNAs regulate and control numerous cellular and physiological processes, such as development, cell proliferation, differentiation, and apoptosis, and they play a role in the pathogenesis of various diseases [1]. The role of miRNAs has been intensively studied in cancer, and many miRNAs play essential roles in cancer-related processes and are consistently either upregulated or downregulated in particular cancer types or specific cancer conditions [2]. Among the miRNAs whose roles in cancer are best documented are the let-7 family, the miR-17-92 cluster (oncomiR-1), and miR-21 [3]. Several miRNAs have also been clinically tested as cancer biomarkers, cancer therapeutics, or targets of cancer treatments [4].

miRNAs are generated from long primary precursors (pri-miRNAs) through a multistage process of miRNA biogenesis, of which the most important steps are (i) excision of the ∼80 nt long hairpin-shaped part (pre-miRNA) catalyzed by the microprocessor complex, whose core is composed of DGCR8 and the nuclease DROSHA (in the nucleus), and (ii) processing of the pre-miRNA by the nuclease DICER1, which cuts off its terminal loop and generates an miRNA duplex (in the cytoplasm). Upon loading into the miRNA-induced silencing complex (miRISC), the miRNA duplex releases one of its strands and uses the other as the guide strand (mature miRNA) that recognizes miRNA targets through sequences usually located in the 3′ untranslated region (3′UTR) sequence of the mRNA.

miRNA precursors are encoded either within protein-coding genes or by independent transcriptional units, also known as miRNA host genes, that are transcribed by RNA polymerase II. The most crucial and conserved part of miRNA transcriptional units is an ∼100 nt long segment encompassing the hairpin-shaped structure of the pre-miRNA, which is commonly referred to and annotated by the HUGO Gene Nomenclature Committee as the miRNA gene. Many miRNA genes occur in clusters of 2–46 genes in one transcriptional unit [5]. Currently, ∼2000 miRNA genes are annotated in the human genome, of which ∼600 are well validated in major miRNA databases, such as miRBase [6] and MirGeneDB [7].

Despite the enormous interest in noncoding RNAs, especially miRNAs, very little is known about the genetic variability of miRNA-encoding genes, including common polymorphisms, hereditary germline mutations, and cancer somatic mutations in these genes. Nonetheless, a few well-documented cases of mutations in miRNA genes are responsible for Mendelian diseases and are recurrently found in cancer. Examples include mutations in *MIR184* [8–10] and *MIR96* [11, 12], which are associated with hereditary eye diseases and nonsyndromic hearing loss, respectively; germline and somatic mutations and deletions of the *MIR15A/MIR16-1* cluster in CLL [13]; and somatic mutations in *MIR142* [summarized in [14]] in different hematologic malignancies, particularly in lymphomas (reviewed in [15]). Additionally, numerous single-nucleotide polymorphisms (SNPs) are located within miRNA genes, some of which (e.g. *MIR125A*, *MIR146A*, *MIR502*, and *MIRLET7*s) are associated with different diseases or disease-related conditions [summarized in [15]]. These variants may affect various aspects of miRNA gene function, including miRNA target recognition (if a mutation occurs in the seed sequence), the miRNA level, the miRNA 5p/3p strand balance, or isomiR predominance [15]; however, functional studies have focused mainly on mutations in seed sequences that affect target recognition. Some effects of mutations are a direct consequence of miRNA sequence changes, but others may result from mutation-induced changes in the structure and stability of miRNA precursors, which affect miRNA biogenesis and processing.

Interest in the noncoding genome has increased with the widespread use of whole-genome sequencing (WGS), but its analysis is still very limited due to the lack of dedicated tools. The limitations concern both computational/statistical methods that allow for identifying, annotating, and interpreting genetic variants in noncoding sequences and experimental techniques, including dedicated sequencing approaches. For example, no such tools are available for noncoding elements, as whole-exome sequencing (WES) is used for coding sequences, enabling targeted sequencing of all coding exons in one experiment. The cost of WGS is still high, strongly limiting its applications in terms of the number of analyzed samples and depth of coverage, which is usually relatively low for WGS, hampering some applications.

We developed whole-miRNome sequencing (WMS), a new next-generation sequencing (NGS)-based approach enabling the targeted sequencing of all miRNA genes complemented by a panel of protein-coding genes that play a role in miRNA biogenesis and function (miRNA biogenesis genes), to address the limitations of the currently used techniques and fill the methodological gap in miRNA gene analysis. We demonstrated the utility of WMS by sequencing several hundred DNA samples, with very high coverage (>700×), of different types and qualities, including high-quality samples extracted from fresh cell cultures, as well as highly degraded, low-quality samples extracted from archival formalin-fixed paraffin-embedded (FFPE) cancer samples. As a result, we detected 2016 mutations, including 581 constitutional mutations in cancer cell lines and 1435 somatic mutations in cancer samples. The robustness of the detected mutations was confirmed through various validation methods. A total of 879 (61%) cancer somatic mutations were located in miRNA genes, including highly validated and cancer-related miRNA genes. The mutations were located in different functional elements of the genes. The analysis revealed the over-occurrence and functional enrichment of mutations in some miRNA genes, which may suggest roles for these genes in cancer. Finally, we demonstrated the utility of WMS in identifying copy number alterations (CNAs, i.e. deletions or duplications/amplifications). In addition to well-known cancer drivers, many identified CNA hotspots also encompass genes encoding cancer-related miRNAs.

## Materials and methods

### Patient sample collection

This study utilized genetic material extracted from pairs of cancer and adjacent normal tissues (or, in some cases, matching blood samples). A total of 286 pairs of tissues were collected: at the Franciszek Lukaszczyk Oncology Center, Department of Molecular Oncology and Genetics, Bydgoszcz, Poland [154 FFPE lung cancer adenocarcinoma samples (referred to as LUN), 33 FFPE colon cancer (COL) samples, and 20 ovarian cancer (OVA) samples]; at the Medical University of Gdansk, Poland [49 fresh frozen ovarian cancer (OVA) samples with matching control blood samples]; at the Center of Oncology, Maria Sklodowska-Curie Memorial Institute, Cracow Branch, Poland [6 fresh frozen renal carcinoma (REN) samples]; and at the Department of Plastic Surgery, St. Josef Hospital, Catholic Clinics of the Ruhr Peninsula, Essen, Germany [24 fresh basal cell carcinoma (BCC) tissues placed in RNAlater (Qiagen, Hilden, Germany) and stored at −80°C]. The samples were collected after signed informed consent forms were obtained from the patients. This study was approved by the Bioethics Committee of Poznan University of Medical Sciences, Poland (03 August 2018), and was conducted in accordance with the local institutional bioethical committees and the Declaration of Helsinki.

### WMS panel design

The WMS panel is designed to capture a specific set of targets encompassing 1849 miRNA genes, the exons of 28 genes involved in miRNA biogenesis and function, and 10 well-known lung cancer-driver genes (or their hotspot-containing exons). The genomic coordinates of all the targeted regions are listed in Supplementary Table S1. The WMS probes were designed in the Agilent SureDesign web portal, if possible, utilizing validated probes from the SureSelect Human All Exon V6 + UTR r2 enrichment panel (Agilent Technologies, Santa Clara, CA, USA) or designing new probes according to the general SureDesign recommendation. Twenty-nine miRNA genes were excluded because of their location in repetitive regions, segmental duplications, and/or low-complexity sequences. For most of the miRNA biogenesis genes, the WMS panel covers the entire coding sequence (CDS), 5′UTR, and 3′UTR sequences. The 3′UTRs of some genes were not covered (*AGO1*, *LIN28A*, *LIN28B*, *SMAD4*, and *TNRC6A*) or were covered partially (*AGO2*, *AGO3*, and *SRSF3*). Although probes were designed against the GRCh19 genome assembly, all downstream analyses were performed, and the results are presented against GRCh38. WMS is available from Agilent SureSelect Custom target capture under design ID: 3115731.

### NGS and data analysis

Before library preparation, all DNA samples were quantified using a NanoDrop One (Thermo Scientific, Waltham, USA) and Qubit fluorometer 3.0 (Invitrogen Carlsbad, CA, USA) [Qubit dsDNA HS Assay (Life Technologies, Carlsbad, USA)]. The quality/integrity of each sample was assessed with our routine in-lab procedure, which was based on an image of the sample (50–400 ng) captured after agarose gel electrophoresis, on a 4-point scale where 3 indicates DNA mainly in high-molecular-weight fragments (upper band), 2 indicates a smear of different molecular sizes, 1 indicates DNA mostly in the low-molecular-weight fragments (lower band), and 0 indicates no visible signal. Most of the libraries were prepared with 200 ng of DNA using the SureSelectXT Target Enrichment System for Illumina Paired-End Multiplexed Sequencing Library Kit (Agilent Technologies, Santa Clara, CA, USA) according to the manufacturer’s recommendations. For a small group of samples (11 pairs), the SureSelectXT HS kit was used. The samples were sequenced on the Illumina (San Diego, CA, USA) HiSeq 4000 platform (180 samples) and NovaSeq 6000 platform (414 samples) in 100 bp paired-end read mode. Library preparation, enrichment, and targeted sequencing were performed at Macrogen, Inc. (Seoul, Republic of Korea). Demultiplexing of the sequencing reads was performed with Illumina bcl2fastq (v 2.20). Low-quality reads were filtered out, and adapters were removed with AdapterRemoval v.1.5.4. The paired-end reads were aligned to the human reference genome (version GRCh38/hg38) using the Burrows–Wheeler Aligner (BWA-mem) [16]. Duplicate reads were removed, and sequencing metrics were collected with the Picard tools package (v 2.19.0). Indel realignments and base quality score recalibration were performed with GATK version 4.1.2.0. SAM to BAM conversion was performed with SAMtools (v 1.2) [17].

### Sample fingerprinting

We verified the matching of the analyzed pairs of tumor and normal samples by comparing their genotypes based on an analysis of 10 independent (unlinked) SNPs located in distant miRNA and miRNA biogenesis genes [18], [19] and with a minor allele frequency (MAF) > 0.1 in the general population according to the 1000 Genomes Project (Supplementary Table S2). Seven sample pairs showing signs of mispairing were excluded from further analysis.

### Somatic mutation calling and filtration

Somatic variants were called with MuTect2 version 4.1.0.0 [20] with the default parameters in the tumor–normal mode and with the gnomAD resource to filter germline variants based on the population allele frequency. We used FilterMutectCalls to filter out variants for which the alternative read median base quality and mapping quality were too low (--min-median-base-quality 20 and --min-median-mapping-quality 30). Additionally, we filtered out common SNPs (dbSNP release 153) and mutations with a variant allele frequency (VAF) < 0.05 to further increase the reliability of the identified mutations (and avoid the identification of uncertain mutations). Finally, we removed variants with an SEQQ > 20, indicating that alternative alleles are not sequencing errors, and GERMQ quality > 20, indicating that the alternative alleles are not germline variants.

The miRNA gene mutations were annotated using a modified miRMut protocol [21] and a set of in-house Python scripts described previously [22]. A few miRNA genes assigned in miRBase v.22 as “dead entries” were removed from the analysis. Finally, we analyzed 1817 miRNA genes defined as sequences encoding hairpin-shaped pre-miRNAs flanked upstream and downstream by 25 nucleotides, as described previously [14, 23], and roughly corresponding to/overlapping the miRNA gene designated by the HUGO Gene Nomenclature Committee. All mutations were defined according to the HGVS nomenclature at the transcript (for miRNA- and protein-coding genes) and/or protein levels (for protein-coding genes), and the effects of mutations in protein-coding genes were predicted using the Ensembl Variant Effect Predictor (VEP) tool [24]. Each miRNA gene mutation was assigned a functionally weighted miRMut mutation score [14, 23]. The score is based on the localization of mutations in miRNA gene functional motifs/subregions, i.e. seed sequence (guide-strand only) (2×); miRNA duplex (1.5×); functional protein-binding motifs; DROSHA/DICER1 cleavage sites (1.5×); and other locations, including flanks and the miRNA precursor apical loop (1×). For the visualization of mutations in protein-coding genes on the gene maps, we used ProteinPaint from St. Jude Children’s Research Hospital—PeCan Data Portal [25]. The protein domains visualized on gene maps were positioned according to UniProt [26].

### Validation of mutations with Sanger sequencing

We selected 43 mutations of different types, different VAFs, and from different tumors to verify the sequencing results. For each of the selected mutations, we designed a pair of primers flanking the putative mutation site and generating a PCR amplicon of ∼400 nt in length (Supplementary Table S3). PCR was performed according to the manufacturer’s recommendations (GoTaq DNA polymerase, Promega, Madison, WI, USA). All the PCR products were purified using the EPPIC Fast Kit (A&A Biotechnology, Gdynia, Poland) and sequenced in two directions using the BigDye v3.1 Kit (Applied Biosystems, Foster City, CA, USA). The sequencing products were separated with capillary electrophoresis on an ABI PRISM 3130xl (Applied Biosystems, Foster City, CA, USA).

### Structure predictions and web servers used

The secondary structures of miRNA precursors were predicted using mfold software [27] with default parameters and visualized with the VARNA application [28]. Three-dimensional structural predictions of pre-miRNA stem-loop structures were generated using RNAComposer software [29] with default parameters and visualized in PyMOL (Schrödinger, LLC, New York, NY, USA).

The prediction of targets for wild-type and mutant miRNAs was performed with the use of TargetScan Custom (v.5.2) [30], and the overlap between the wild-type and mutant targets was visualized as Venn diagrams [31].

### Identification of potential cancer drivers with OncodriveFML

The identification of potential cancer drivers was performed with OncodriveFML (v.2.4.0) [32] across all samples (pancancer) and separately for individual cancer types using the CADD score [33]. Additionally, the analysis of BCC samples was complemented with the use of the DANN score [34], which should provide enhanced accuracy in predicting the functional consequences of noncoding variants. The signatures were computed by cancer type as a classifier, with the statistical method set to “arithmetic mean,” including indels. The recommended *q*-value 0.25 threshold was used for significant results.

### Analysis of CNAs

CNAs in individual samples were identified using CNVkit [35] by comparing them with a reference created from normal samples for each tumor type separately. The segmentation files generated by CNVkit were subsequently analyzed with GISTIC2.0 [36] (with WMS-targeted positions indicated as markers) to identify hotspots of deletions and amplifications across the following sets of cancer samples: LUN, OVA, COL, and BCC. In the case of LUN, only samples sequenced with SureSelectXT (*n* = 140) were included; 11 samples sequenced with the SureSelectXT HS kit were excluded. The following parameters were used: threshold for copy number amplifications and deletions, 0.2; confidence level to calculate the region containing a driver, 0.9; broad-level analysis; and the arm-level peel-off method to reduce noise. We excluded centromeric regions that were highly variable and rich in repetitive sequences to improve the reliability of the analysis and reduce false-positive signals. Although the sex assignment was correctly performed by the tools used, for clarity, the final results are shown for the human autosomal genome.

### Cancer cell line analysis

The cells were grown in appropriate media to a confluency of ∼80%, and DNA was isolated using an AllPrep DNA/RNA/miRNA Universal Kit (QIAGEN, Hilden, Germany). WMS was performed as described above using 200 ng of DNA and quality filtering parameters. We reported all sequence variants identified by Mutect2 that had a VAF > 0.05 and did not overlap with common SNPs (dbSNP release 153).

## Results

### Development of the WMS panel

**Figure 1.**
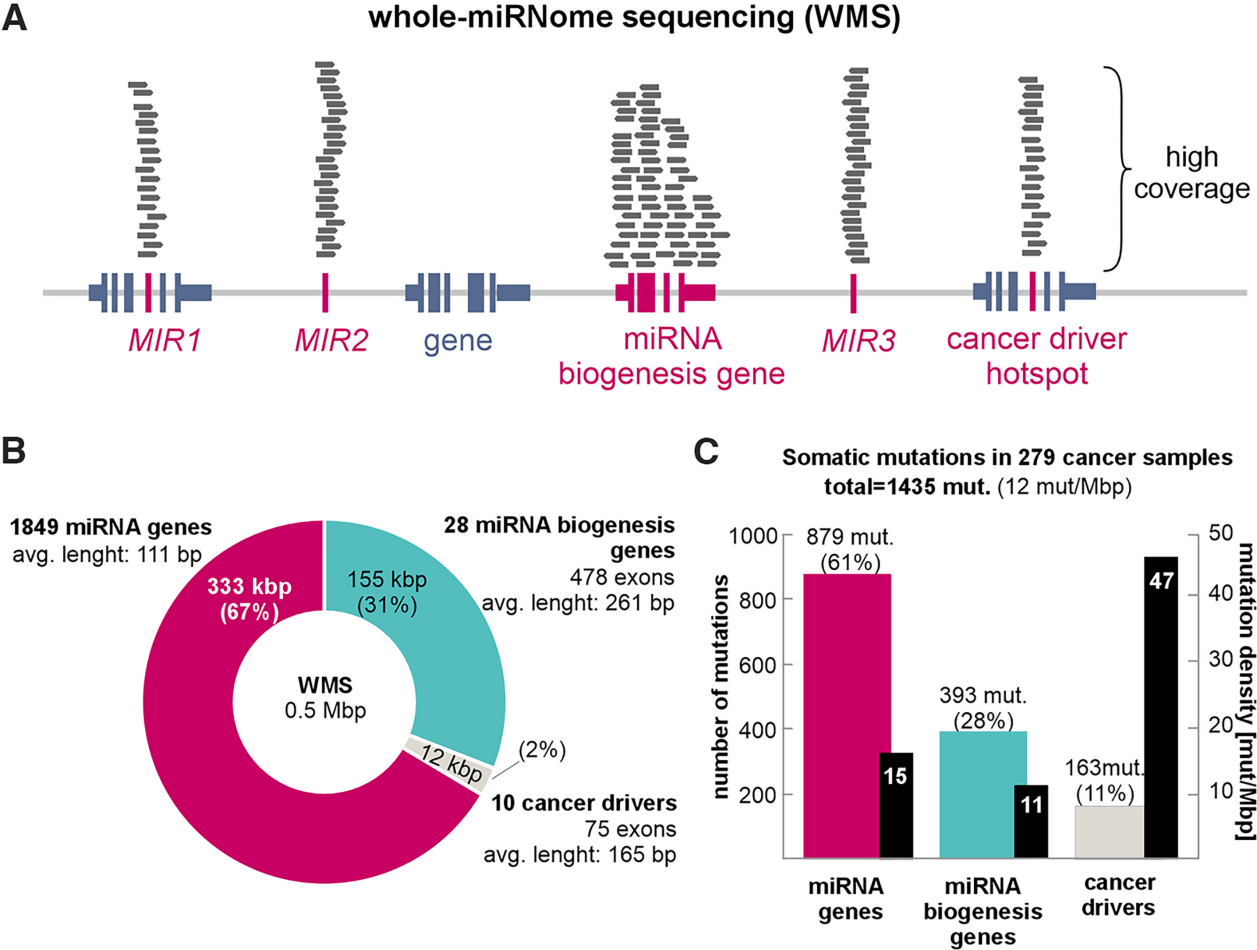
WMS panel design and performance. (**A**) Schematic representation of regions (marked in magenta) enriched by WMS. (**B**) The proportion and number of regions targeted by WMS: miRNA genes (magenta), miRNA biogenesis genes (turquoise), and cancer-driver genes (gray). (**C**) The number (color bars, left axis) and density (black bars, right axis) of somatic mutations identified in miRNA genes, miRNA biogenesis genes, and cancer-driver regions in 279 cancer samples with WMS; mut/Mbp represents the average number of mutations per million nucleotides per sample.

In the framework of this study, we designed the first WMS panel. The panel covers 96% of all (1849/1917) and 99% of validated (499/505) miRNA genes annotated in miRBase (22 release) [6], as well as 99% (503/507) of mRNA genes annotated in MirGeneDB [7]. The small fraction of miRNA genes not covered by the panel consists mainly of poorly validated genes, often embedded in low-complexity repeat-rich sequences or duplicated genomic regions. Consistent with the HUGO Gene Nomenclature Committee, we defined sequences encoding pre-miRNAs (as annotated in miRBase) with their adjacent flanks (∼20 nt) as miRNA genes. Additionally, we supplemented the panel with 28 miRNA biogenesis genes, including genes that play a role in (i) the transcription of primary miRNA precursors (pri-miRNAs) [*SMAD4*], (ii) pri-miRNA to pre-miRNA processing in the nucleus [*FUS*, *SRSF3* (SRP20), *DROSHA*, *DGCR8*, *DDX5* (P68), *DDX17* (P72), and *GSK3B*], (iii) the export of pre-miRNA to the cytoplasm [*XPO5* (EXP5) and *RAN*], (iv) pre-miRNA processing and miRNA maturation [*DICER1* (DICER), *TARBP2* (TRBP), *PRKRA* (PACT), *ADAR*, *KHSRP* (FUBP2, KSRP), *LIN28A*, *LIN28B*, *ZCCHC11* (TUT4), *ZCCHC6* (TUT7), and *DIS3L2*], and (v) miRNA–target recognition/interaction and execution of downstream silencing effects [*AGO1*, *AGO2*, *AGO3*, *AGO4*, *GEMIN4*, *MOV10*, *FMR1*, and *TNRC6A* (GW182)] [as in [22]]. Finally, we included 10 well-known cancer-related genes (or their hotspot-containing exons) listed as most frequently mutated in lung cancer (*BRAF*,*EGFR*,*ERBB2*,*HRAS*,*KRAS*,*MAP2K1*,*MET*,*NF1*,*NRAS*, and *RIT1*) [37] in the panel to serve as internal controls for highly mutated regions (Fig. 1A). In total, the WMS panel encompasses 499 590 bp covered/baited by 16 302 probes (Agilent Tier 1), including 333 174 bp (11 778 probes) of miRNA genes and 166 416 bp (4524 probes) of miRNA biogenesis- and cancer-related gene exons (Fig. 1B). The size of the regions roughly corresponds to the number of mutations detected, except for a higher frequency of mutations detected in the cancer-driver regions, which served as positive control (Fig. 1C).

### WMS performance

We performed WMS on a total of 580 samples to test the performance of the panel: (i) 279 (tumor/normal) pairs of cancer samples, including 151 lung adenocarcinoma (LUN), 68 ovarian carcinoma (OVA), 31 colorectal cancer (COL), 23 skin BCC, and 6 REN samples, and (ii) 22 established cancer cell lines. Most (*n* = 202) of the cancer sample pairs were extracted from FFPE blocks, but some (*n* = 77) were extracted from fresh (RNAlater-preserved) or fresh frozen tissues (fresh tissue, FT). DNA from cancer cell lines (*n* = 22) was extracted directly after cell harvest. As FFPE samples are inherently of low quality at various stages of degradation, before the analysis, the integrity of the DNA samples was briefly determined with our internal laboratory-established procedure on a 4-point scale (from 3–highest quality to 1–lowest quality and 0–not detectable on an agarose gel). The matching of tumor/normal sample pairs was confirmed by SNP fingerprinting (see the “Materials and Methods” section).

**Figure 2.**
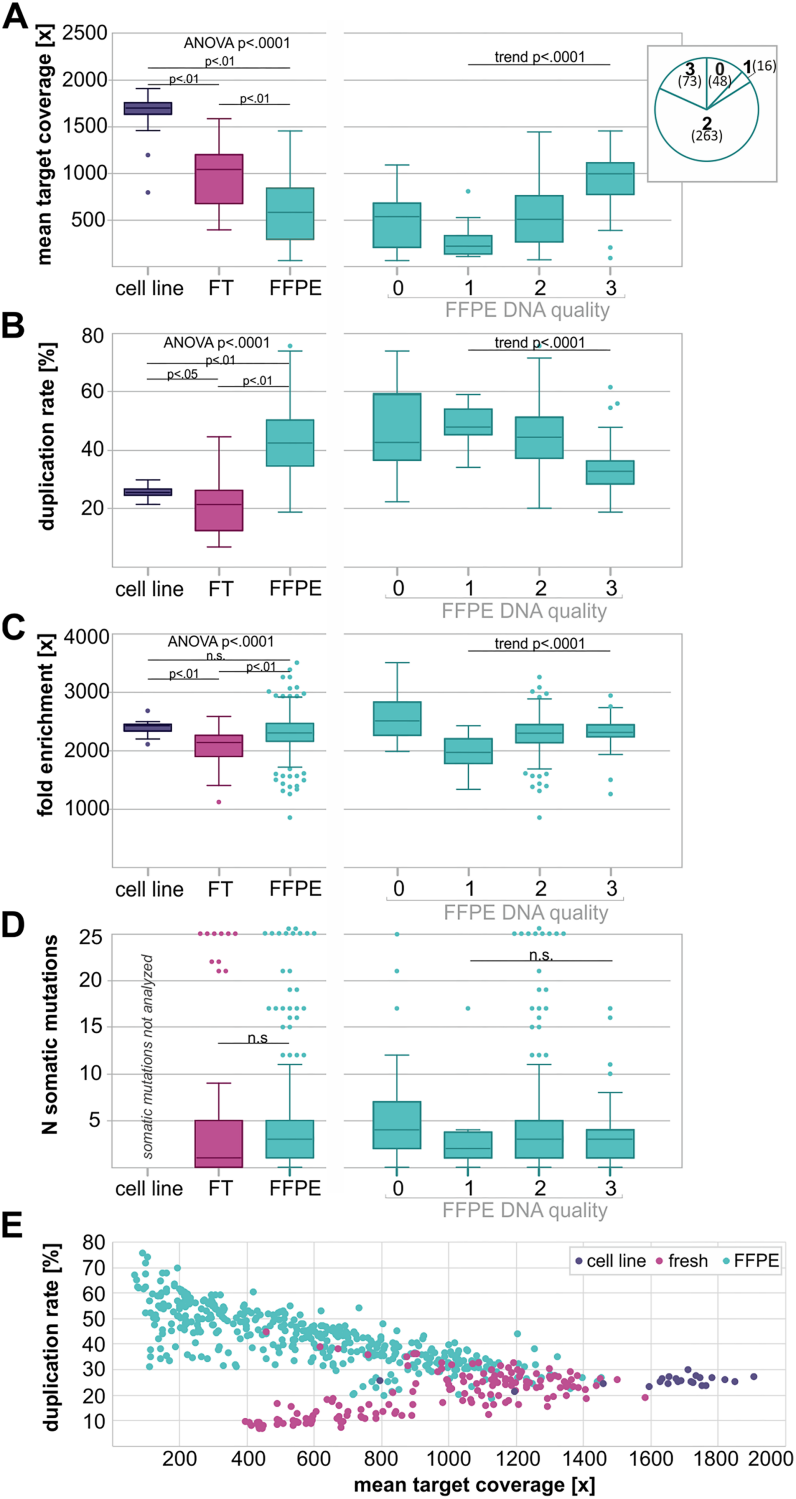
Sequencing metrics and performance of WMS. On the left, Tukey’s box and whisker plots show the distributions of the mean sequencing coverage (**A**), fold enrichment (**B**), duplication rate (**C**), and number of somatic mutations (**D**) in DNA samples extracted from the cell lines, freshly preserved tissue (FT), and FFPE-preserved samples (on the left). On the right, FFPE samples were additionally divided into 4 DNA integrity/quality categories (see the “Materials and methods” section). The proportions of FFPE samples in the particular integrity categories are shown in the inset (pie chart) in panel (A). One-way ANOVA with post-tests or the ANOVA for trend *P*-values for categories 1 to 3 are indicated above the graphs. (**E**) A scatter plot showing the relationship between the mean sequencing coverage and duplication rate of the analyzed samples (dots); the color of the dots indicates the sample type, as indicated in the graph.

With some minor exceptions, libraries were prepared with 150–200 ng of DNA and sequenced to obtain 30 million (M) total reads per sample (3 Gbp). The essential characteristics of WMS performance, i.e. target coverage, duplication rate, target fold enrichment, and number of detected somatic mutations, are shown in Fig. 2A–D and Supplementary Table S4. The mean sample coverage of all samples averaged 732× (range 60–1906×) and, as expected, was somewhat higher for high-quality samples extracted from cell lines (average 1644×) than for archival fresh and fresh frozen tissues and FFPE samples (Fig. 2A). Additionally, FFPE samples with higher integrity scores presented higher coverage than those with lower integrity scores (Fig. 2A). The differences in coverage may be at least partially explained by the duplication rate, which, as shown in Fig. 2B and E, is lower in high-quality samples and roughly inversely correlated with coverage. Despite these differences, the coverage of the vast majority (90%) of samples, including DNA extracted from FFPE samples, was > 200×, which is much higher than that of standard WES or WGS experiments. Even though the miRNome panel is relatively small at ∼0.5 Mb (∼6.200× smaller than the human genome), the baited region has been enriched (over a genomic background) by 2248× on average, and the enrichment did not differ substantially between the sample types (Fig. 2C), indicating the robustness of WMS.

We called somatic mutations with the Mutect2 algorithm and processed them with the miRMut protocol [21]. In 279 cancer samples, we identified a total of 1435 somatic mutations, of which 879 (61%) were located in miRNA genes, 393 (28%) were located in miRNA biogenesis genes, and 163 (11%) were located in control cancer-driver genes (Fig. 1C, Table 1, and Supplementary Table S5). Despite some dependence of the WMS performance parameters on DNA quality (above), we did not observe any effect of DNA quality on the number of detected mutations (Fig. 2D).

**Table 1.** Summary of mutations identified in the analyzed cancer types

	All samples	LUN	OVA	COL	BCC	REN
N pairs	279	151	68	31	23	6
total N of mut.	1435	715	277	240	199	4
N (%) mutated samples	227 (81)	133 (88)	43 (63)	26 (84)	22 (96)	3 (50)
N (%) substitutions	1312 (91)	651 (91)	256 (92)	214 (89)	188 (94)	3 (75)
N miRNA mut.	879	426	164	141	145	3
N (%) mutated samples	187 (67)	116 (77)	30 (44)	16 (52)	22 (96)	3 (50)
N (%) substitutions	825 (94)	395 (93)	155 (95)	133 (94)	140 (97)	2 (67)
N biogenesis mut.	393	184	85	72	51	1
N (%) mutated samples	120 (43)	66 (44)	25 (37)	12 (39)	16 (70)	1 (17)
N (%) substitutions	348 (89)	169 (92)	74 (87)	54 (75)	50 (98)	1 (100)
N cancer mut.	163	105	28	27	3	-
N (%) mutated samples	116 (42)	83 (54)	13 (19)	18 (58)	2 (9)	-
Sample type	-	FFPE	FFPE, FT	FFPE	FT	FT

### WMS validation

First, we resequenced (with Sanger sequencing) 43 mutations in both miRNA and protein-coding genes to estimate the fraction of false-positive mutations. The analysis confirmed 42 mutations, indicating a very low (2%) fraction of potential false-positive mutations. Next, we compared the frequency of mutations in well-established lung adenocarcinoma driver genes in our LUN samples and the previous large-scale study of lung adenocarcinoma reported by TCGA [37]. The analysis revealed that 32% of the samples had mutations in *KRAS*; 14% in *NF1*; 8% in *EGFR*; 3% in *BRAF*; 1% each in *ERBB2*, *MET*, and *NRAS*; and no mutations in *MAP2K1*, which is consistent with the findings of TCGA study [37] (*r*^2^= 0.932; *P* < .0001) (Supplementary Fig. S1). Moreover, as *EGFR* hotspot driver mutations are biomarkers in cancer treatment and are routinely tested in lung cancer samples, we compared our results with those of previous clinical tests performed on the same samples. As shown in Supplementary Table S6, WMS detected all 12 mutations detected previously using commercial high-sensitivity real-time quantitative polymerase chain reaction (RT−qPCR) assays (6× exon 19 in-frame deletions, 3× exon 21 L858R mutation, 2× exon 21 L861Q mutation, and exon 19 S768I mutation). Additionally, with WMS, we detected one exon 19 in-frame deletion (L747_S752del) that was missed in clinical testing. Finally, for 23 BCC samples, we compared the current WMS results with our previous results [38], in which the same samples were sequenced using a commercial WES platform [SureSelectXT Human All Exon V6 kit (Agilent)]. These two datasets substantially differed in sequencing depth, which was 701× and 183× in the compared region covered by both assays (28 miRNA biogenesis genes) for WMS and WES, respectively. Despite this difference, the results of both WMS and WES were very consistent; WMS identified all 37 mutations detected by WES and additionally detected 2 mutations not detected before. The new mutations could most likely be detected because of the much higher coverage of WMS.

### General characteristics of the somatic mutations identified in miRNA genes

All (*n* = 879) mutations identified in miRNA genes were annotated and characterized using miRMut, a specialized pipeline dedicated to the accurate annotation of genetic variants in miRNA genes developed previously in our laboratory [21]. The mutations were identified in 635 miRNA genes, 242 of which are considered high confidence according to miRBase and/or miRGeneDB, and 53 were classified as cancer-related in the Cancer miRNA Census (CMC) [2] (CMC-miRNA). At least one miRNA gene mutation was detected in 187 (67%) cancer samples, 63 (34%) samples had more than one mutation, and 10 (5%) had more than 10 mutations. The vast majority (93.9%) of the identified mutations were substitutions, 3.2% were deletions, 1.5% were insertions, and 1.5% were dinucleotide substitutions. The frequency and type of mutations in different cancer types are summarized in Table 1.

**Figure 3.**
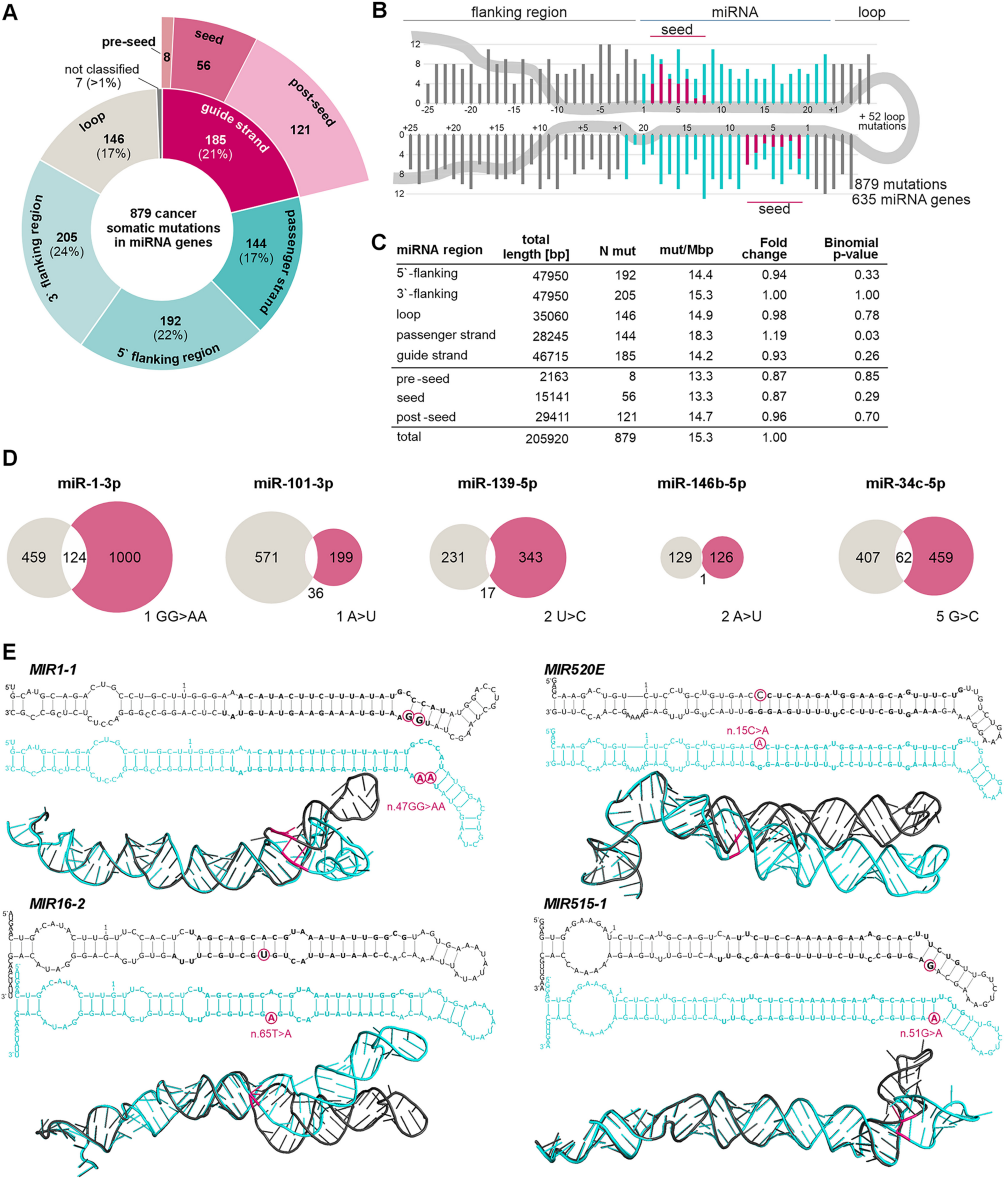
Cancer somatic mutations identified in miRNA genes. (**A**) Distribution of the mutations among the miRNA precursor subregions. The mature miRNA sequence was further divided into pre-seed—the first nucleotide of the mature miRNA, seed—the 2nd–8th nucleotides of the miRNA, and post-seed—the sequence downstream of the seed. (**B**) Distribution of the identified somatic mutations along the consensus miRNA precursor sequence. The miRNA duplex positions are indicated in turquoise, the seed regions in magenta (only of the mature miRNA strand), the flanking regions, and the apical loop in gray. If present, mutations localized beyond position 22 in mature miRNAs are shown cumulatively at position 22. As the size and structure of the apical loops differ substantially among miRNA precursors, the plot shows only the first and last five positions of the loop region; the number of the remaining loop mutations is indicated within the loop. (**C**) Summary and statistics of the mutations identified in subregions of miRNA precursors, including subregion size and mutation density [mut/Mb—an average number of mutations per million nucleotides per sample]. (**D**) Venn diagrams showing the effect of representative mutations located in miRNA seeds on the number of predicted (TargetScan v.5.2 Custom) targets; as examples, miRNAs annotated in the CMC were selected [2]. The gray and magenta circles indicate the targets predicted for the wild-type and mutant seeds, respectively. The mutated seed positions and the nucleotide changes are shown next to the diagrams. (**E**) The effect of exemplary mutations on the secondary 2D (upper images) and spatial 3D (lower images) structures of miRNA precursors, generated with mfold and RNAComposer software, respectively. Wild-type and mutant structures are shown in black and turquoise, respectively; miRNA duplexes are indicated in bold; and mutation positions are indicated in magenta.

We more closely examined the localization of the identified mutations by categorizing them into different miRNA precursor subregions (Fig. 3A) and superimposing them onto the consensus miRNA precursor structure (Fig. 3B). As shown in Fig. 3B and C, the mutations were more or less evenly distributed throughout the entire miRNA precursor sequence (average density 15.3 mut/Mb). No evidence (binomial distribution) of an unequal distribution of mutations was observed between the functional miRNA gene subregions (ranging from 13 to 18 mut/Mb). Among the identified mutations, 185 (21%) were located in mature miRNAs (guide strands), with 56 being in seed regions, which are the most crucial part of the miRNA gene. Seed mutations severely affect miRNA–target recognition (examples in [15]). As shown in Fig. 3D for 5 exemplary miRNA seed mutations identified in CMC-miRNAs (miR-1–3p, miR-101–3p, miR-139–5p, miR-146b-5p, and miR-34c-5p), the seed mutations severely affect the spectrum of the predicted target genes, which is consistent with our previous results [14, 23]. Another 66 mutations were located in DROSHA and DICER1 cleavage sites, including 47 mutations that altered GYM, a motif essential for efficient DICER1 cleavage [39]. The prominent examples of such mutations are n.45G>A in *MIR30D* and n.41G>T in *MIR320A*, which dramatically lower the GYM score from 73 to 18 and from 58 to 11, respectively; thus, these mutations are likely to decrease the precursor processing efficiency. Additionally, 40 mutations were located in sequence motifs recognized by regulatory RNA-binding proteins, which may affect the accuracy and efficiency of miRNA precursor processing (Supplementary Table S5).

In addition to affecting the sequence of functional elements, most miRNA gene mutations affected the structure and stability of miRNA precursors. Examples of such mutations are shown in Fig. 3E.

### Identification of miRNA genes enriched with cancer mutations

**Figure 4.**
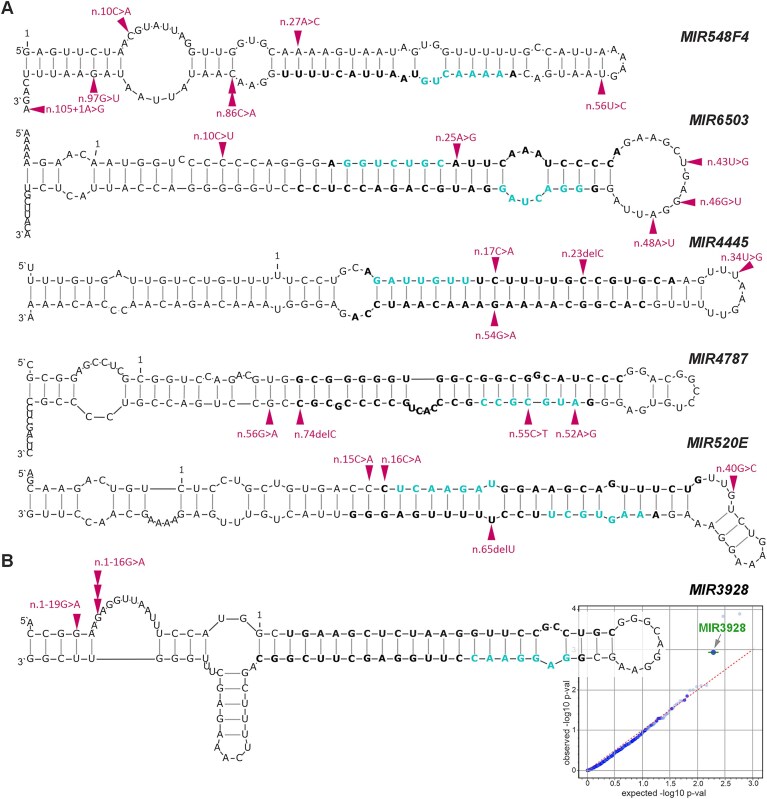
Recurrently mutated miRNA genes. (**A**) Localization of mutations in miRNA genes with 5 or more cancer somatic mutations or with 5.5 or higher functionally weighted miRMut mutation scores. The structures were modeled with mfold for reconstructed pre-miRNAs extended by 25 nt upstream and downstream flanking sequences. Magenta arrowheads indicate the mutation positions, bold fonts indicate miRNA duplexes, and blue fonts indicate seeds of mature miRNAs. The first nucleotides of the miRNA precursors, according to miRBase are indicated at position 1. The positions are used for mutation annotation according to the HGVS nomenclature. (**B**) Positions of mutations in the *MIR3928* gene, showing the most significant functional bias in the OncoDriveFML analysis. All of which was identified as a potential cancer driver. All mutations were identified in BCC. The quantile−quantile (QQ) plot shows the results of OncodriveFML analysis (with the CADD score) visualized as the distribution of expected (*x*-axis) and observed (*y*-axis) *P*-values corresponding to functional mutation enrichment in particular miRNA genes. The analysis was performed based on the mutations detected in all cancer samples (pancancer analysis). The green color indicates genes defined as significant (*q* < 0.25) according to the OncodriveFML recommendation.

To demonstrate the usefulness of the WMS, we performed several analyses of the detected miRNA gene mutations. First, we performed the identification of miRNA genes with a higher frequency of mutations or functionally weighted mutations than expected by chance. The analysis showed that 5 miRNA genes were significantly overmutated (binomial distribution *P*< .0003), with ≥ 5 mutations or ≥ 5.5 functionally weighted miRMut mutation scores. Among them were *MIR548F4* with 7 mutations (miRMut score of 7.5), *MIR6503* with 5 mutations (miRMut score of 5.5), *MIR4445* with 4 mutations (miRMut score of 5.5), *MIR4787* with 4 mutations (miRMut score of 6.5), and *MIR520E* with 4 mutations (miRMut score of 6) (Fig. 4A and Supplementary Table S7).

Next, we analyzed the mutations identified in the miRNA genes with OncodriveFML, a tool for identifying potential cancer drivers [32]. OncodriveFML takes advantage of functional mutation scores (e.g. CADD, a tool for scoring the deleteriousness of mutations [33]) for the identification of functional mutation biases (enrichment over random chance) in tested regions. OncodriveFML is recommended for both coding and noncoding regions. The cumulative analysis across all cancer samples (pancancer analysis) identified one miRNA gene, *MIR3928* (Fig. 4B), with functional mutation bias. Further analysis of individual cancer types revealed that the bias is specific to BCC, a cancer type in which all*MIR3928* mutations occur (Supplementary Fig. S2A). The functional mutation bias of *MIR3928* in BCC was also confirmed using DANN (Supplementary Fig. S2B), an alternative score for annotating the pathogenicity of genetic variants with improved performance in noncoding regions [34]. *MIR3928* was mutated with 4 mutations in 4 BCC samples. The mutations were located in two nearby positions in the 5′ flanking region, n.1–16G>A and n.1–19G>A (Fig. 4A). Three of the mutations (with VAF > 20%) were analyzed and confirmed by Sanger sequencing. Other miRNA genes identified with OncodriveFML signal in individual cancer types include *MIR548AG2* in BCC (in total, 4 mutations, miRMut score of 5), *MIR3138* in LUN (in total, 3 mutations, miRMut score of 3.5), and *MIR507* in OVA (in total, 3 mutations, miRMut score of 3) (Supplementary Fig. S2). It has to be noted, however, that all of these genes may be considered only candidate driver genes, and their true cancer-driver character would require confirmation in an extensive functional study.

### Identification of mutations in miRNA biogenesis genes and potential cancer drivers

**Figure 5.**
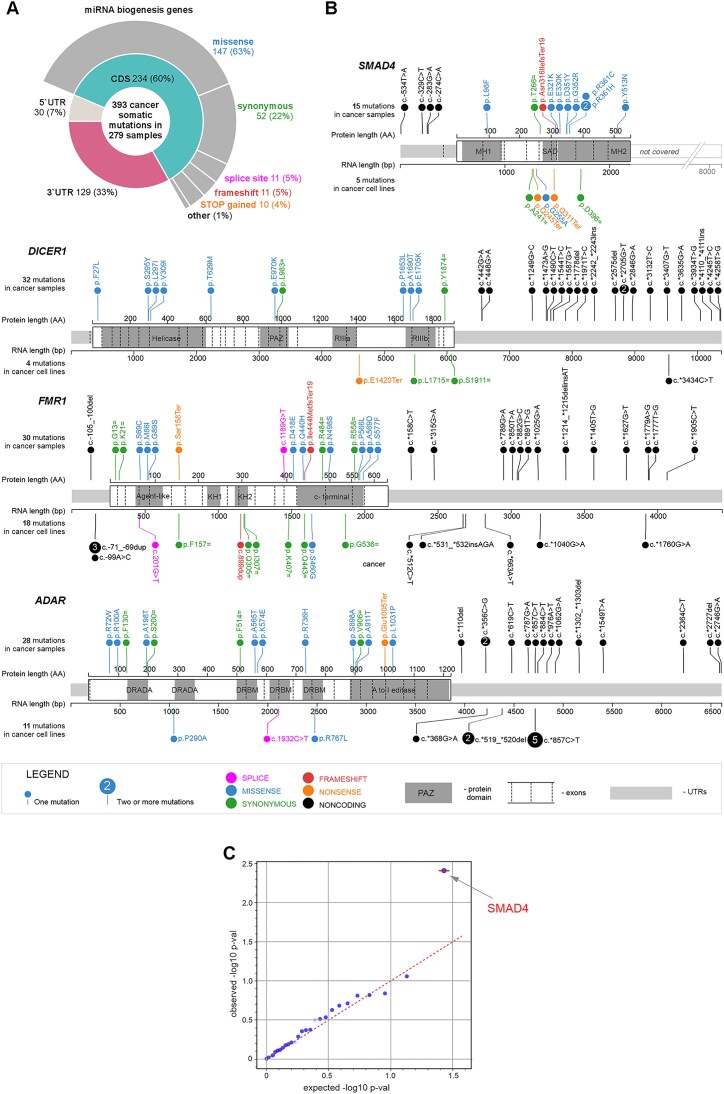
Summary of somatic mutations detected in miRNA biogenesis genes. (**A**) A doughnut chart showing the types (outer circle) and localization (subregions; inner circle) of mutations detected in miRNA biogenesis genes. (**B**) Maps of the most frequently mutated miRNA biogenesis genes. The cancer somatic mutations and constitutional mutations detected in cancer cell lines are indicated as lollipops above and below the maps, respectively; the mutation types are indicated in colors (see the legend). (**C**) QQ plot showing the results of the OncodriveFML analysis (with the CADD score) visualized as the distribution of expected (*x*-axis) and observed (*y*-axis) *P*-values corresponding to the functional enrichment bias of coding mutations in miRNA biogenesis genes based on the mutations detected in all cancer samples (pancancer analysis). The red color indicates genes defined as highly significant (*q* < 0.1) according to the OncodriveFML recommendation.

In the panel of 28 miRNA biogenesis genes, we identified 393 mutations, including 30 (7%) in 5′UTRs, 234 (60%) in CDSs, and 129 (33%) in 3′UTRs. Among the CDS mutations were 147 missense mutations, 52 synonymous mutations, and 32 definitive deleterious mutations, including 11 frameshift, 10 nonsense, and 11 splice-site mutations (Fig. 5A and Supplementary Table S5). At least one mutation was detected in 120 (43%) samples. The frequency of mutated samples ranged from 17% in REN samples to 70% in BCC samples (Table 1), roughly corresponding to the mutational burden in the analyzed cancer types. Among the most highly mutated genes were *DICER1* with 32 mutations (of which 11 were localized in the CDS), *FMR1* with 30 mutations (16 in the CDS), *ADAR* with 28 mutations (14 in the CDS), *DROSHA* with 27 mutations (22 in the CDS), and *SMAD4* with 15 mutations (11 in the CDS) (Fig. 5B and Supplementary Table S5).

The analysis of the CDS mutations using OncodriveFML (Fig. 5C) revealed a very strong and distinct signal for *SMAD4* as a potential cancer driver (*q* < 0.1). This result further confirms the reliability of the mutations detected by the WMS panel, as *SMAD4* is a very well-known tumor suppressor gene that plays a role and is frequently mutated in pancreas, colon, and other intestinal duct cancers, as well as in lung adenocarcinoma [22, 40, 41]. Consistent with previous results, most *SMAD4* mutations identified in our study were found in LUN (8 CDS mutations) and COL (4 CDS mutations), and most of them clustered in the MH2 domain, including the well-recognized hotspot mutations p.R361C, p.R361H, and p.G352R, which are known to disrupt SMAD4–SMAD2 heterotrimer formation, consequently affecting SMAD4 transcription factor activity [22, 42–44]. Another known cancer-driver mutation identified in our study is p.E1705K in *DICER1*, which was detected in a COL sample. The mutation was previously observed in various cancers, predominantly in uterine/endometrial cancer [45, 46]. The p.E1705K mutation is located in the catalytic center of the RNase IIIb (RIIIB) domain at the metal ion-binding residue, and it was shown to affect the proper processing of miRNA precursors [22, 47, 48].

### Copy number alteration (CNA) analysis

**Figure 6.**
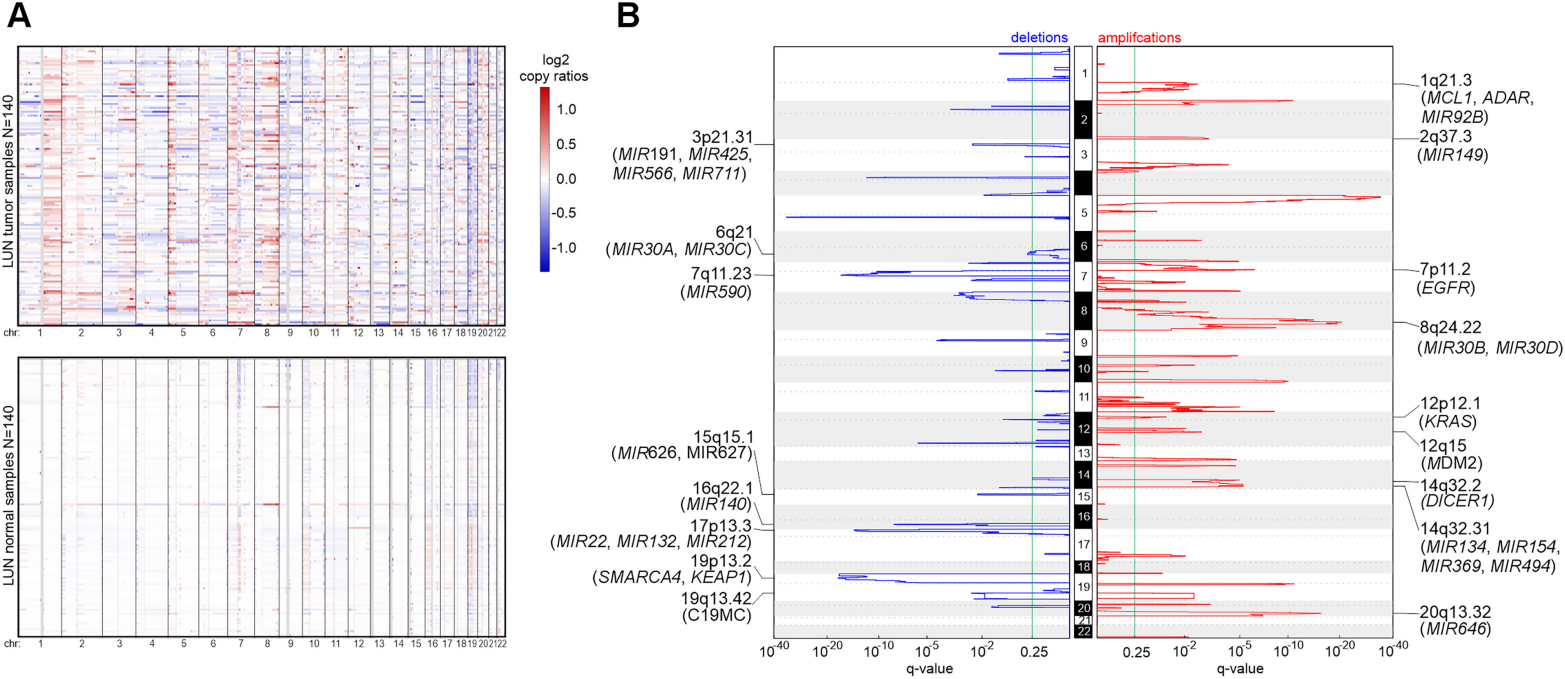
CNA analysis using WMS data from LUN samples. (**A**) Heatmap displaying individual CNAs in tumor (top panel) and normal (bottom panel) samples. Blue and red indicate deletions and duplications/amplifications, respectively; the color intensity indicates the log_2_ of the copy-number amplitude as shown in the legend, according to CNVkit. Each row represents an individual sample; columns represent chromosomes (indicated below). (**B**) A plot indicating the chromosomal locations (vertical axis) of recurrent amplification (red) and deletion (blue) peaks detected by GISTIC2.0. The height of the peaks (horizontal axes) indicates the significance (*q*-value) of the region with an amplification/deletion recurrence. The green line indicates the recommended significance threshold, *q* = 0.25. The selected deleted and amplified regions/genes are indicated on the graphs.

Despite the low number of targeted regions (*n* ∼ 2000) and the limited number of sequenced cancer samples, we attempted to use WMS for a chromosome-arm-level CNA analysis and the identification of hotspot deletions and amplifications. As shown in Fig. 6A for LUN and in Supplementary Fig. S3 for COL, OVA, and BCC, the analysis allowed the detection of numerous somatic CNAs in cancer samples. The analysis also correctly predicted (based on the copy number of sex chromosomes) the sex of the patients from whom the analyzed samples were collected. As expected, CNAs did not occur or were very rare in normal samples (Fig. 6A and corresponding panels in Supplementary Fig. S3). Since normal samples were extracted from the same tissue types as the corresponding cancer samples and were treated in the same way, the level of CNAs observed in the normal samples may roughly reflect the level of false-positive results. The patterns of recurrent/hotspot CNAs were generally consistent with those previously reported for the corresponding cancers [37, 49, 50] (Fig. 6B and Supplementary Fig. S3). For example, we identified 22 LUN hotspot deletions and 27 amplifications (Fig. 6B). Among the hotspot amplifications were the regions encompassing *EGFR* (ch7p11),*KRAS* (ch12p12), and *MCL1* (ch1q21), which are well-recognized cancer drivers that play crucial roles and are known to be amplified in lung adenocarcinoma [37]. Many of the identified CNAs also included well-known cancer-related miRNA genes, including *MIR92B* (ch1q21), *MIR30B* (ch8q24), and *MIR30D* (ch8q24), whose frequent amplifications in lung adenocarcinoma have been reported previously [51], and many others that have not been reported previously (Fig. 6B). Among the miRNA genes located in hotspot deletions are *MIR140*, *MIR132*, *MIR212*,and*MIR22*, which act as tumor suppressors [52–54], and the entire chromosome 19 cluster (C19MC), which consists of 46 miRNA genes involved in the regulation of the cell cycle and proliferation [55], including *MIR520E*, which we identified as frequently mutated in the LUN samples (Fig. 4A). The results of the CNA analysis of other cancer types are shown in Supplementary Fig. S3. Notably, consistent with our previous study [38] performed using WES, we identified the recurrent deletion of chromosome 9q and the recurrent duplication/amplification of chromosome 9p in the BCC samples. Chromosome 9q includes the tumor suppressor *PTCH1*, the most crucial driver gene for BCC, and a cluster of 3 miRNA genes (*MIRLET7A*, *MIRLET7B*, and *MIRLET7F*), members of the tumor suppressor *let-7* family [56], whereas chromosome 9p includes the *CD274* (encoding PD-L1), *CD273* (encoding PD-L2), and *JAK2* oncogenes. All chromosome 9 alterations detected in individual samples were consistent with our previous study, in which they were independently confirmed with MLPA [38]. Detailed lists of all identified CNAs are shown in Supplementary Table S8.

### WMS of cancer cell lines

Cell lines are frequently utilized for the functional characterization of various miRNAs, and miRNA gene mutations can impact these analyses. Therefore, as an additional example of a WMS application, we characterized 22 established cancer cell lines (listed in Table 2). In total, we identified 412 mutations in miRNA genes (75% of which were unique), 138 mutations in miRNA biogenesis genes, and 31 in the control cancer-driver genes (the distribution of mutations in the most frequently mutated genes is shown in Fig. 5B). The number of miRNA gene mutations ranged from 91 in SCC-25 (tongue squamous cell carcinoma) to 4 in SKNMC (bone metastatic neuroblastoma). Among the miRNA genes mutated in the cell lines, 50 were annotated in the CMC, including *MIR21*, *MIR146B*, *MIR150*, and *MIR345*, with mutations in the miRNA duplex; *MIR142*, *MIR200C*, and *MIR335*, with mutations in the flanking sequences; and *MIR101-1*, *MIR26A1*, *MIR30A*, *MIR106B*, *MIR221*, *MIR152*, *MIR224*, and *MIR27A*, with mutations in the apical loops of miRNA precursors. The list of all mutations in miRNA and miRNA biogenesis genes (Supplementary Table S9) may serve as a resource for selecting an appropriate cell line for the functional analysis of specific miRNAs.

**Table 2.** Summary of mutations found in miRNA and miRNA biogenesis genes identified in the selected cancer cell lines

cell line	type	N mut. in miRNA genes (N mutated miRNA genes)	examples of mutated miRNA genes	N mut. in miRNA biogenesis genes; total/coding (N mutated genes)
A549	lung adenocarcinoma	15 (15)	*MIR200C* ^CMC^, MIR3130-1, MIR3622A	4/2 (4)
CAL-27	tongue squamous cell carcinoma	9 (8)	MIR146B ^CMC^, MIR6077	1/1 (1)
FaDu	pharynx squamous cell carcinoma	14 (14)	MIR1302-2,*MIR4528*	0/0 (0)
H1299	non-small cell lung carcinoma	17 (15)	MIR3196, MIR2053, MIR548M, MIR6869	5/1 (4)
H358	bronchioalvealar adenocarcinoma	18 (18)	*MIR5010*	12/5 (7)
HEK293T	kidney epithelial cells	9 (8)	MIR198, MIR4430,*MIR548AI-2*	4/2 (3)
HeLa	cervical cancer	16 (13)	MIR5696, MIR609, MIR548AI-2, MIR7157	3/1 (3)
HT-29	colon adenocarcinoma	12 (12)	MIR4767, MIR4321	2/2 (2)
LN-18	glioblastoma	8 (7)	MIR4449, MIR2116, MIR654, MIR6842	2/1 (2)
MCF7	breast adenocarcinoma	8 (8)	*MIR152* ^CMC^, MIR1294, MIR8084	7/4 (7)
MDA-MB-231	breast adenocarcinoma	18 (15)	MIR4636, MIR3945, MIR4463, MIR1255A	3/1 (3)
MeWo	malignant melanoma	47 (14)	MIR3163, MIR513C, MIR517B, MIR6840, MIR515, MIR432	15/10 (10)
LN229	glioblastoma	13 (13)	MIR3686, MIR3978, MIR6839	4/2 (2)
SCC25	tongue squamous cell carcinoma	91 (63)	*MIR16-1* ^CMC^, *MIR142*^CMC^, MIR21^CMC^, *MIR30A*^CMC^, *MIR106B*^CMC^, *MIR101-1*^CMC^, *MIR26A1*^CMC^, *MIR221*^CMC^, MIR345^CMC^, MIR193B, MIR191, MIR190A	41/34 (14)
SH-SY-5Y	neuroblastoma	10 (10)	MIR520H, MIR6859-1	0/0 (0)
SK-BR-3	breast adenocarcinoma	14 (12)	*MIR224* ^CMC^, MIR602, MIR6811, MIR4463	2/0 (1)
SK-MEL-28	malignant melanoma	16 (15)	*MIR150* ^CMC^, MIR1285-2, MIR3196, MIR4472-2	3/1 (3)
SK-MES-1	lung squamous cell carcinoma	12 (10)	*MIR378E*	4/2 (4)
SK-N-MC	neuroblastoma	4 (4)	MIR3690-1, MIR6864	2/0 (2)
SK-OV-3	ovary adenocarcinoma	36 (34)	*MIR27A* ^CMC^, *MIR345*^CMC^, MIR4716, MIR509-1, MIR5692B, MIR6727	7/4 (6)
T47D	breast ductal carcinoma	8 (8)	*MIR335* ^CMC^, MIR509-3	10/3 (6)
U-2 OS	osteosarcoma	17 (17)	*MIR3977*, MIR4463	7/3 (5)

bold—miRNA genes with mutations in seed; underline—miRNA genes with mutations in miRNA duplex; and ^CMC^—miRNA genes annotated in CMC.

## Discussion

The WMS developed in this study is the first sequencing system enabling the parallel sequencing of all miRNA genes. We sequenced several hundred samples of various cancer types and a panel of established cancer cell lines to show the potential of WMS. As a result, we identified nearly 1500 somatic mutations in cancer samples and over 500 constitutive mutations in the established cancer cell lines. Approximately two-thirds of these mutations were identified in miRNA genes, with the remaining mutations in miRNA biogenesis genes. At least one miRNA gene mutation occurred in 67% of the cancer samples. However, this percentage varied between cancer types, with the highest percentage occurring in BCC and LUN and the lowest in REN and OVA, which is consistent with the general mutational burden in these cancer types [57]. Although proportional across cancer types, the fractions of samples with mutations in miRNA genes were somewhat higher in this study than in TCGA samples [14]. This discrepancy is likely due to the much higher sequencing depth in this study and the fact that the sequencing of TCGA samples did not cover all the miRNA genes.

The reliability of the detected mutations was verified through various methods, including analyzing mutations using alternative molecular methods and comparing mutations detected in coding regions to data previously published by large-scale and well-recognized projects such as TCGA. All of these analyses showed nearly perfect agreement or even the superiority of WMS over other methods, thus confirming the robustness of WMS in detecting mutations with various VAFs in samples of different types, including low-quality archival FFPE samples.

The analysis of identified mutations revealed that miRNA gene mutations are evenly distributed along the gene sequence and occur in all miRNA precursor subregions, including flanking sequences, the miRNA duplex, and the apical loop. Some mutations were identified in most crucial functional elements of miRNA genes, such as the seed sequence, DROSHA and DICER1 cleavage sites, or binding motifs of the regulatory RNA-binding proteins. A previous functional analysis of phenotypically neutral SNPs located in miRNA genes showed that the majority of sequence variants in miRNA genes affect the accuracy and effectiveness of miRNA processing [19]. Therefore, the expectation that a substantial number of mutations identified in this study also have the potential to affect the function of miRNA genes is justified. Although few in number, examples of mutations in miRNA genes associated with human diseases, including monogenic Mendelian diseases, indicate that sequence variants can impact miRNA genes by disrupting various aspects of their function, including the efficiency of miRNA processing/miRNA level, the 5p/3p strand ratio, the shift in DROSHA/DICER1 cleavage sites generating alternative isomiRs, and the efficiency of the recognition and silencing of target genes (summarized and discussed [15]). These effects are anticipated to be obtained either by the direct impacts of mutations changing the key functional sequences of miRNA genes, such as seed or protein-binding sites, or by the influence of the mutations on the structure and stability of miRNA precursors. Nonetheless, since most mutations in the cancer genome are randomly occurring neutral variants, only a small fraction of mutations detected in miRNA genes may play a role in carcinogenesis, regardless of their impacts on miRNA genes. The most potent of these variants may be mutations in well-known cancer-related miRNA genes, such as *MIR16-2*, *MIR143*, or *MIR155*, or mutations recurring in specific miRNA genes.

Although the analysis of miRNA gene mutations with OncodriveFML had low statistical power, it identified several miRNA genes with enriched signals of mutation functionality, with *MIR3928* presenting the most substantial evidence. The mutations in *MIR3928* occur specifically in BCC, with 4 mutations in 4 different samples. All the mutations are located in the 5p flanking region, including one singleton mutation and one hotspot mutation (occurring in 3 samples), which are located 21 and 18 nucleotides upstream of the pre-miR-3928 sequence, respectively. Computational modeling indicated that mutations, especially hotspot mutations, locally modify the structure and decrease the stability of the miR-3928 precursor. *MIR3928* is a well-validated miRNA gene (miRBase); however, it has not been strongly implicated in cancer, except for one recent study suggesting a role for miR-3938 in glioma [58]. However, *MIR3928* should only be considered a candidate cancer driver, and the oncogenic nature of its mutation should be interpreted cautiously. Proving the oncogenic/driver nature of mutations in a gene would require extensive functional and clinical analyses, as well as testing the gene in additional larger panels of samples, which are beyond the scope of our study.

In comparison, the strongest candidate cancer driver identified among the miRNA biogenesis genes was *SMAD4*. This well-known tumor suppressor plays a role and is commonly mutated in various solid tumors, particularly in pancreatic and other intestinal duct cancers [22, 40, 41]. The identification of *SMAD4* confirms the reliability of the collected data and the analysis performed here. SMAD4 is a well-characterized transcription factor that functions as a heterotrimer with other SMAD family members and regulates the transcription of various genes, including miRNA genes [59, 60]. Mutations in *SMAD4*, especially in the MH2 domain, prevent the interaction of SMAD4 with other SMADs and, thus, the formation of a functional complex [22].

As shown in the study, WMS can identify various types of mutations in different types of human genomic samples, including germline and somatic mutations, whether rare or common, and with different VAFs. The WMS data may also be used to identify chromosome-arm-level CNAs. Specifically, WMS may be used to identify mutations in diseases or conditions with “hidden heritability,” in which mutations in coding genes and other types of genetic variations routinely tested for diseases have been excluded. Examples of such cases may include mutations in *MIR96* [11, 12] and *MIR204* [61] identified in patients with hereditary conditions, nonsyndromic hearing loss, and retinal dystrophy, in which no mutations in known genes associated with the conditions were identified. Another application of WMS may be the in-depth characterization or screening of mutations in an miRNA gene or group of genes in which mutations have already been detected or suspected. These genes may include *MIR14*2, for which somatic mutations have been identified in various blood cancers [14], or *MIR15A and MIR16-1*, which are commonly deleted and/or mutated in chronic lymphocytic leukemia [13]. WMS may also be used to identify new genes responsible for cancer predisposition. For example, although many genes have already been identified and are being tested for breast cancer predisposition, new genes are still being sought to explain all suspected heritable cases. To date, all these analyses have ignored noncoding genes, including miRNA genes. Next, WMS may be used to prescreen samples or cell lines before selecting them for the functional study of miRNA or miRNA-related processes to avoid using a model with a mutation in the miRNA of interest. Finally, WMS may be used as a complementary test to miRNA expression profiling, as, on the one hand, miRNA gene mutations may affect miRNA levels, and, on the other hand, miRNAs with unchanged levels may not be functional due to mutations, e.g. in the seed sequence.

The additional value of our study is the catalog of miRNA gene mutations in established cancer cell lines. Some of these mutations are located in extensively studied cancer-related genes (e.g. *MIR16-1* and *MIR21* in SCC25 cells, *MIR152* in MCF7 cells, *MIR150* in SK-MEL-28 cells, or *MIR345* in SK-OV-3 cells). This information may be essential and should be considered when selecting a cell line for an miRNA functional study or when interpreting the functional results in a particular cell line. Similarly, mutations in miRNA biogenesis genes (e.g. nonsense mutations in *DICER1* in H358 cells and *SMAD4* in CAL27 and HT-29 cells) may be considered when performing relevant experiments.

To the best of our knowledge, WMS is the only approach allowing targeted sequencing of all miRNA genes. Although some WES platforms may overlap with or coincidentally cover some miRNA genes, sequencing the entire miRNome using these platforms would not be practical and would be far from complete. For example, the Human All Exon V8 + NCV Agilent platform, extended for detecting noncoding variants, covers only 98 (∼5%) miRNA genes. Therefore, thus far, the WGS method has been the only option for sequencing miRNA genes. In contrast to WGS, WMS allows the sequencing of all miRNA genes at a much lower cost. The primary benefit of using WMS is the much lower cost of sequencing miRNA genes in the human genome than the only available alternative, WGS. For example, the cost of our experiment of sequencing 580 samples with ∼700× coverage, including library preparation and sequencing, was $133 400 ($230 per sample). The cost may be further substantially reduced by using newer machines and applying higher indexing/multiplexing of samples. It allows for sequencing with a much higher coverage or analysis of a much larger number of samples. An additional advantage of the WMS system is the relatively small amount of DNA sample needed. In this study, we used 150–200 ng for sequencing, while the minimum amount recommended for WGS is approximately 10× higher (2 μg). The low sample consumption substantially increases the applicability of WMS for low-quantity samples, which are often cancer samples but also other human samples. Finally, in our experiment, we demonstrated the applicability of WMS for different types of DNA samples, including very low-quality, highly degraded FFPE cancer samples.

In conclusion, this study involved the development of WMS, a tool for the targeted sequencing of all human miRNA genes. We used WMS to sequence over 300 cancer samples, as well as cell line samples, and identified 1291 mutations in miRNA genes. The high reliability of the detected mutations and the robustness of WMS were confirmed by various methods. However, interpreting the functional consequences of sequence variants in noncoding sequences, such as miRNA genes, remains challenging. Improving this area will require the collection of more data, the development of new computational and statistical approaches, and the expansion of genetic knowledge. Tools such as WMS can assist in gathering relevant data, ultimately contributing to understanding the consequences of mutations in miRNA genes.

## Supplementary Material

gkaf812_Supplemental_Files

## Data Availability

All the data generated or analyzed during this study are included in the published paper and associated Supplementary files. The sequencing data were deposited in the National Center for Biotechnology Information (NCBI, http://www.ncbi.nlm.nih.gov/) under the Sequence Read Archive (SRA) accession number PRJNA1235613. WMS is available from Agilent SureSelect Custom target capture, under design ID: 3115731.
