# Supplementary material for: Whole-miRNome sequencing: a panel for the targeted sequencing of all human miRNA genes
Source: Nucleic Acids Res. 2025 Aug 27;53(16):gkaf812. doi: 10.1093/nar/gkaf812 (PMC12390761; doi:10.1093/nar/gkaf812)
Supplement: gkaf812_Supplemental_Files [file gkaf812_supplemental_files.zip › SUPPLEMENTARY FIGURES.docx]

**SUPPLEMENTARY FIGURES**

**Supplementary Figure S1.** Comparison of the mutation frequency in lung adenocarcinoma driver genes identified in this study in LUN samples (n=151) and TCGA lung adenocarcinoma project (n=230) (37). The Pearson correlation coefficient (R^2^) and p-value are indicated on the graph.

**Supplementary Figure S2. . OncoderiveFML analysis of miRNA genes, performed separately for mutations identified in BCC, LUN, OVA, and COL.** The QQ plots show the distribution of expected (x-axis) and observed (y-axis) p-values corresponding to functional mutation bias calculated with (A) CADD and (B) DANN scores. The green and red colors indicate genes defined as significant (q<0.25) and highly significant (q<0.1), respectively, according to the OncodriveFML recommendation.

**Supplementary Figure S3. CNA analysis using WMS data from the OVA, COL, and BCC samples.** The figure scheme is shown in Figure 6.

**SUPPLEMENTARY TABLES**

Supplementary_Table_S1_WMS_coordinates.xlsx

Supplementary_Table_S2_Fingerprinting.xlsx

Supplementary_Table_S3_Primers.xlsx

Supplementary_Table_S4_WMS_metrics.xlsx

Supplementary_Table_S5_List_of_mutations_cancer_samples.xlsx

Supplementary_Table_S6_EGFR_qPCR.xlsx

Supplementary_Table_S7_Hotspot_miRNAs.xlsx

Supplementary_Table_S8_CNAs.xlsx

Supplementary_Table_S9_List_of_mutations_cell_lines.xlsx
